# IBT-V02: A Multicomponent Toxoid Vaccine Protects Against Primary and Secondary Skin Infections Caused by *Staphylococcus aureus*

**DOI:** 10.3389/fimmu.2021.624310

**Published:** 2021-03-10

**Authors:** Hatice Karauzum, Arundhathi Venkatasubramaniam, Rajan P. Adhikari, Tom Kort, Frederick W. Holtsberg, Ipsita Mukherjee, Mark Mednikov, Roger Ortines, Nhu T. Q. Nguyen, Thien M. N. Doan, Binh An Diep, Jean C. Lee, M. Javad Aman

**Affiliations:** ^1^Integrated BioTherapeutics, Rockville, MD, United States; ^2^Division of HIV, Infectious Diseases, and Global Medicine, Department of Medicine, University of California, San Francisco, San Francisco, CA, United States; ^3^Division of Infectious Diseases, Department of Medicine, Brigham and Women's Hospital and Harvard Medical School, Boston, MA, United States

**Keywords:** *S. aureus*, toxoid vaccine, skin infection, pore-forming toxins, superantigens

## Abstract

*Staphylococcus aureus* causes a wide range of diseases from skin infections to life threatening invasive diseases such as bacteremia, endocarditis, pneumonia, surgical site infections, and osteomyelitis. Skin infections such as furuncles, carbuncles, folliculitis, erysipelas, and cellulitis constitute a large majority of infections caused by *S. aureus* (SA). These infections cause significant morbidity, healthcare costs, and represent a breeding ground for antimicrobial resistance. Furthermore, skin infection with SA is a major risk factor for invasive disease. Here we describe the pre-clinical efficacy of a multicomponent toxoid vaccine (IBT-V02) for prevention of *S. aureus* acute skin infections and recurrence. IBT-V02 targets six SA toxins including the pore-forming toxins alpha hemolysin (Hla), Panton-Valentine leukocidin (PVL), leukocidin AB (LukAB), and the superantigens toxic shock syndrome toxin-1 and staphylococcal enterotoxins A and B. Immunization of mice and rabbits with IBT-V02 generated antibodies with strong neutralizing activity against toxins included in the vaccine, as well as cross-neutralizing activity against multiple related toxins, and protected against skin infections by several clinically relevant SA strains of USA100, USA300, and USA1000 clones. Efficacy of the vaccine was also shown in non-naïve mice pre-exposed to *S. aureus*. Furthermore, vaccination with IBT-V02 not only protected mice from a primary infection but also demonstrated lasting efficacy against a secondary infection, while prior challenge with the bacteria alone was unable to protect against recurrence. Serum transfer studies in a primary infection model showed that antibodies are primarily responsible for the protective response.

## Introduction

*Staphylococcus aureus* (SA) is an opportunistic pathogen responsible for a wide range of clinical infections, ranging from superficial skin lesions, deep-seated abscesses, and osteomyelitis to life threatening sepsis, endocarditis and pneumonia (1, 2). SA has a propensity for acquiring resistance to antibiotics, with epidemics of penicillin-, methicillin- and vancomycin-resistant strains occurring since 1940 (3). Methicillin-resistant SA (MRSA) represents ~50% of SA infections in the US (4). Therefore, development of an effective vaccine against various SA diseases is of utmost importance. An effective vaccine can not only have a major impact on public health, but it will also significantly reduce the burden of antimicrobial resistance. Prior efforts for development of vaccines and immunotherapeutics have largely focused on the surface proteins or polysaccharides (ClfA, SdrG, IsdB, MntC, CP5, and CP8) (5, 6). Unfortunately, to-date these vaccines have failed to meet their clinical endpoints in human efficacy trials. Opsonic antibodies represent a reliable correlate of protection for several other vaccines such as pneumococcal, meningococcal and *H. influenzae* vaccines (7). Remarkably, all the SA vaccines advanced into clinic were strong inducers of antibodies that promoted opsonophagocytic activity (8–10), putting in question whether the paradigm of opsonic antibodies as correlate of immunity can be applied to SA. In the case of one of these vaccine candidates, the single component vaccine consisting of the iron-regulated protein IsdB (V710, Merck), the trial was stopped due to safety concerns after ~8,000 patients undergoing cardiothoracic surgery were recruited. Among those patients who developed a SA infection, a higher number of multi-organ failures and death occurred in the vaccine arm compared to placebo (11). A follow up study in a subset of these patients suggested that a combination of immunization with IsdB, SA infection, and the immunological status of the host (reflected in low or undetectable serum IL-2 and IL-17 at the time of vaccination) contributed to the catastrophic outcome (12). Furthermore, both studies in mice (13) and rabbits (14) showed a vaccination induced disease enhancement when whole cell vaccine or crude surface antigens were used for immunization, raising the potential for induction of a deleterious immune response by cell associated antigens.

Over the past two decades great advances have been made in our understanding of the immunological effects of the large number of toxins produced by SA and their role in pathogenesis (15–17). These toxins, in particular the cytolytic and superantigenic toxins, mediate a wide range of immune subversive and tissue destructive functions to support infection (18, 19). The cytolytic toxins comprise the single subunit alpha hemolysin (α-toxin, Hla) and a family of bicomponent toxins, including the closely related Panton-Valentine leukocidin (PVL), leukocidin ED (LukED), gamma hemolysins (HlgAB and HlgCB), as well as phylogenetically more distant leukocidin AB (LukAB; also referred to as LukGH) (18, 20). These cytolysins are involved in disruption of skin and mucosal barriers (Hla, PVL), killing of key innate immune cells at the first line of defense against SA (PVL, LukAB, LukED, HlgA/CB), platelet aggregation (Hla), and lysis of human red blood cells to extract iron for bacterial growth (HlgAB) (20). Superantigens (SAgs), comprising the staphylococcal enterotoxins (SEs such as SEA, SEB, SEC, SED, SEK) and toxic shock syndrome toxin-1 (TSST-1), can cause massive polyclonal activation of T cells leading to toxic shock, induce intoxication and dysregulation of a variety of specialized T cell subsets, and induce anergy and lymphocyte apoptosis (21, 22). Collectively, these toxins create a smoke screen rendering the host unable to mount an effective immune response (23). We hypothesized that vaccination with key staphylococcal toxins can protect against SA infections by protecting tissues and the immune system, thus preventing the complications of disease *via* clinical protection even in the absence of sterile immunity. To this end, we have developed a multicomponent vaccine IBT-V02, comprised of toxoids for Hla (Hla_H35LH48L_), subunits of PVL (LukS_mut9_, LukF_mut1_), LukAB (LukAB_mut50_) and a fusion of three SAgs (TSST-1, SEB, and SEA; TBA_225_) and reported the attenuation and efficacy of individual components or partial combinations in bacteremia, pneumonia, and toxic shock models in mice and rabbits (24–27).

Skin and soft tissue infections (SSTI) caused by SA are of special significance worldwide due to their prevalence, the possibility of other disease complications and the associated costs (28, 29). Skin infections typically include furuncles, carbuncles, impetigo, cellulitis, and skin abscesses (30), and methicillin-resistant SA (MRSA) isolates have been found to play a major role in these infections, particularly clones such as USA100, USA300, ST-80, and USA1000 (31–34). Toxins secreted by these and other strains of *SA* serve as key virulence factors in the pathogenesis of SA skin infections, specifically Hla (35), PVL (36, 37) and SAgs (38, 39). Up to 50% of patients with a primary skin infection can experience one or more repeated bouts of recurrent infection over 1 year, even after successful initial treatment by incision and drainage and antibiotics (40–42). Healthy adults, patients with underlying chronic diseases like diabetes, cancer, vascular disease, eczema, lung disease, infection with human immunodeficiency virus, as well as specific populations (military personnel, athletes, injection drug users, prisoners) are at risk of primary and recurrent skin infections caused by SA (30, 43). Hence there is an urgent need for a vaccine providing protection against SA acute skin infections (SA-ASI) and its recurrence.

Here we report that IBT-V02 can induce a strong neutralizing antibody response in mice and rabbits and provide strong protection against primary and secondary SA-ASI. Importantly, we demonstrate that the vaccine protects in both naïve and immunologically pre-exposed mice, a finding that is relevant to human vaccine development since a large portion of the population is or has been colonized with SA. We further show that the vaccine can be administered during the acute phase of primary infection to protect against a subsequent encounter with SA.

## Results

### Formulation and Characterization of the Multicomponent Toxoid Vaccine

IBT-V02 consists of rationally designed toxoid formulations representative of pore-forming and superantigenic toxins: Hla, PVL, LukAB, SEA, SEB, as well as TSST-1. The Hla toxoid harbors two mutations in the N-terminal domain (H35L and H48L) that renders the protein unable to heptamerize and is thus fully non-toxic (27, 44). We have previously described mutagenesis of the S and F subunits of our PVL toxoid LukS_mut9_ and LukF_mut1_ (24). LukS_mut9_ toxoid contains three mutations (T28F/K97A/S209A) and LukF_mut1_ toxoid harbors a single mutation (K102A). Each of these toxoids are fully attenuated in combination with the wild type or mutant PVL counterpart or non-canonical partners (24). LukAB_mut50_ is a dimeric toxoid form of LukAB with mutations introduced in LukA (D39A) and LukB (R23E) that render the protein unable to form a pore in the plasma membrane of target cells (25). TBA_225_ is a fusion protein of the three superantigen toxoids for TSST-1, SEB and SEA (26). Each of the toxoids are mutated in the MHC Class II binding sites and therefore unable to crosslink T cells and antigen presenting cells, resulting in a loss of superantigenicity. The individual mutants are: TSST-1_L30R/D27A/I46A_, SEB_L45R/Y89A/Y94A_, and SEA_L48R/D70R/Y92A/H225A_. The three mutants are fused together *via* a flexible linker 3x(GGGGS) in the order of TSST-1, SEB, and SEA (26).

**Figure 1:**
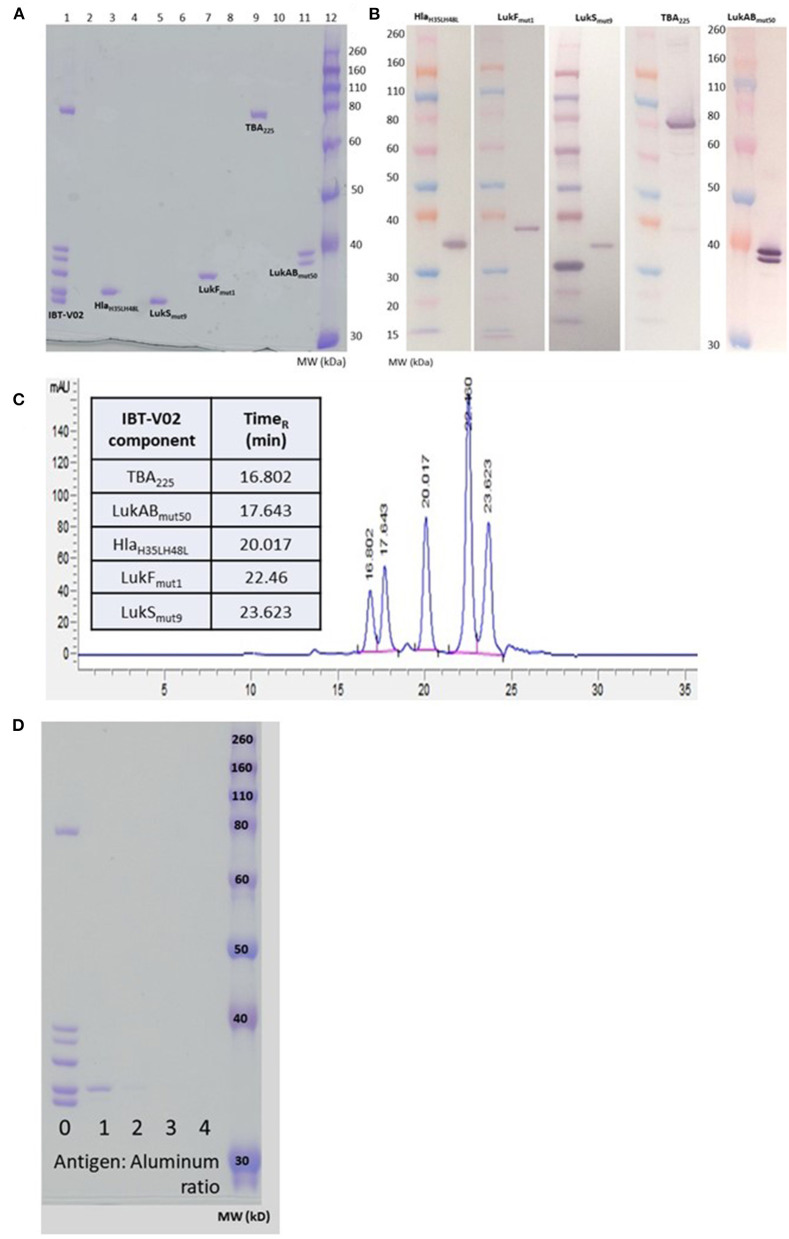
Biochemical characterization of IBT-V02 components. **(A)** SDS PAGE of blended (Lane 1) and individual components (Lanes 3, 5, 7, 9, 11). **(B)** Western Blot analysis and **(C)** SEC-HPLC of individual components. **(D)** SDS PAGE to determine protein adsorption of blended components to Al(OH)_3_.

The individual toxoids were expressed in *E. coli* and purified by multistep column chromatography. The LukAB subunits were expressed from a single plasmid and copurified as a heterodimer. The individual components were blended at equal weight ratios. Figure 1 shows the SDS-PAGE (A) and Western blot (B) analysis of the blended vaccine components and their respective molecular weights. Additionally, using SEC-HPLC, the high level of purity of the individual components could be demonstrated (Figure 1C).

We had previously demonstrated the immunogenicity of the IBT-V02 components in combination with Alhydrogel® (24–26). To optimize the formulation for the multicomponent vaccine we incubated the blended vaccine components with Alhydrogel® at ratios of 1, 2, 3, and 4 (protein:Alhydrogel®) for 60 min at room temperature (RT) followed by centrifugation. To detect any unadsorbed protein in the samples, the supernatants were run on SDS-PAGE and proteins visualized by Coomassie Blue staining. As shown in Figure 1D, a minimum of three-fold excess was needed for complete adsorption by Alhydrogel®.

### Immunogenicity of IBT-V02

**Figure 2:**
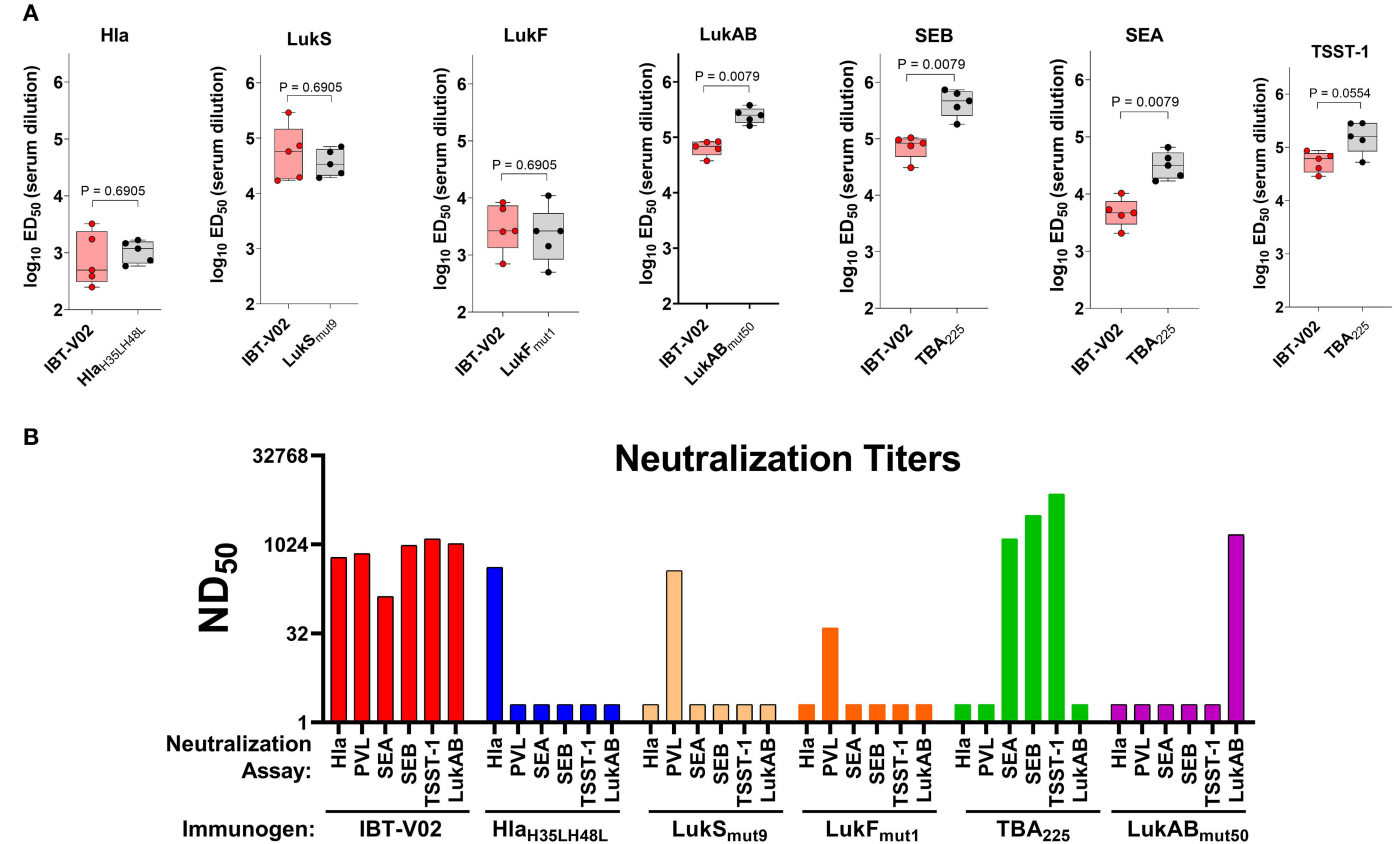
Immunogenicity of IBT-V02 in BALB/c mice, *n* = 5/group. **(A)** Serum IgG binding titers and **(B)** Serum toxin neutralizing titers of mice immunized with blended 5-component toxoid vaccine (IBT-V02) or with each individual toxoid tested against wildtype target antigens Hla, LukS, LukF, LukAB, SEA, SEB, and TSST-1.

Immunogenicity was evaluated in groups of 5 BALB/c mice vaccinated on days 0, 14, and 28 with either IBT-V02 (50 μg/mouse) or individual components (10 μg/mouse) formulated with Alhydrogel® at a ratio of 1:5. Sera were collected on day 42. Individual serum samples were used to determine total antigen specific IgG titers by a multiplex assay developed in house. Pooled sera from each group were also used to determine the neutralization titers against target antigens Hla, PVL, LukAB, SEA, SEB, TSST-1. Antibody titers were determined as the effective dilution of the sera that produced 50% maximal response (ED_50_). As shown in Figure 2A, IgG titers to Hla, LukS, or LukF generated by immunization with IBT-V02 were not significantly different from the titers induced by immunization with the individual toxoids, suggesting that blending the 5 toxoid components did not have a negative impact on the response to these antigens. Whereas, IBT-V02 induced robust serum antibody titers to LukAB (ED_50_ titers ranging from 37,608 to 84,051), SEA (ED_50_ titers from 2,070 to 10,352), SEB (ED_50_ titers from 30,898 to 104,426), and TSST-1 (ED_50_ titers from 28,681 to 86,701), these titers were significantly lower compared to titers generated to the individual toxoids. Nonetheless, the neutralizing antibody titers of serum samples were comparable between IBT-V02 and individual components (Figure 2B). Of the two PVL components, LukS_mut9_ appeared to make a larger contribution to PVL neutralization than LukF_mut1_ (Figure 2B). Overall, the data showed that IBT-V02 induces strong binding and neutralizing antibody titers in mice.

### IBT-V02 Protects Against Primary SA-ASI

**Figure 3:**
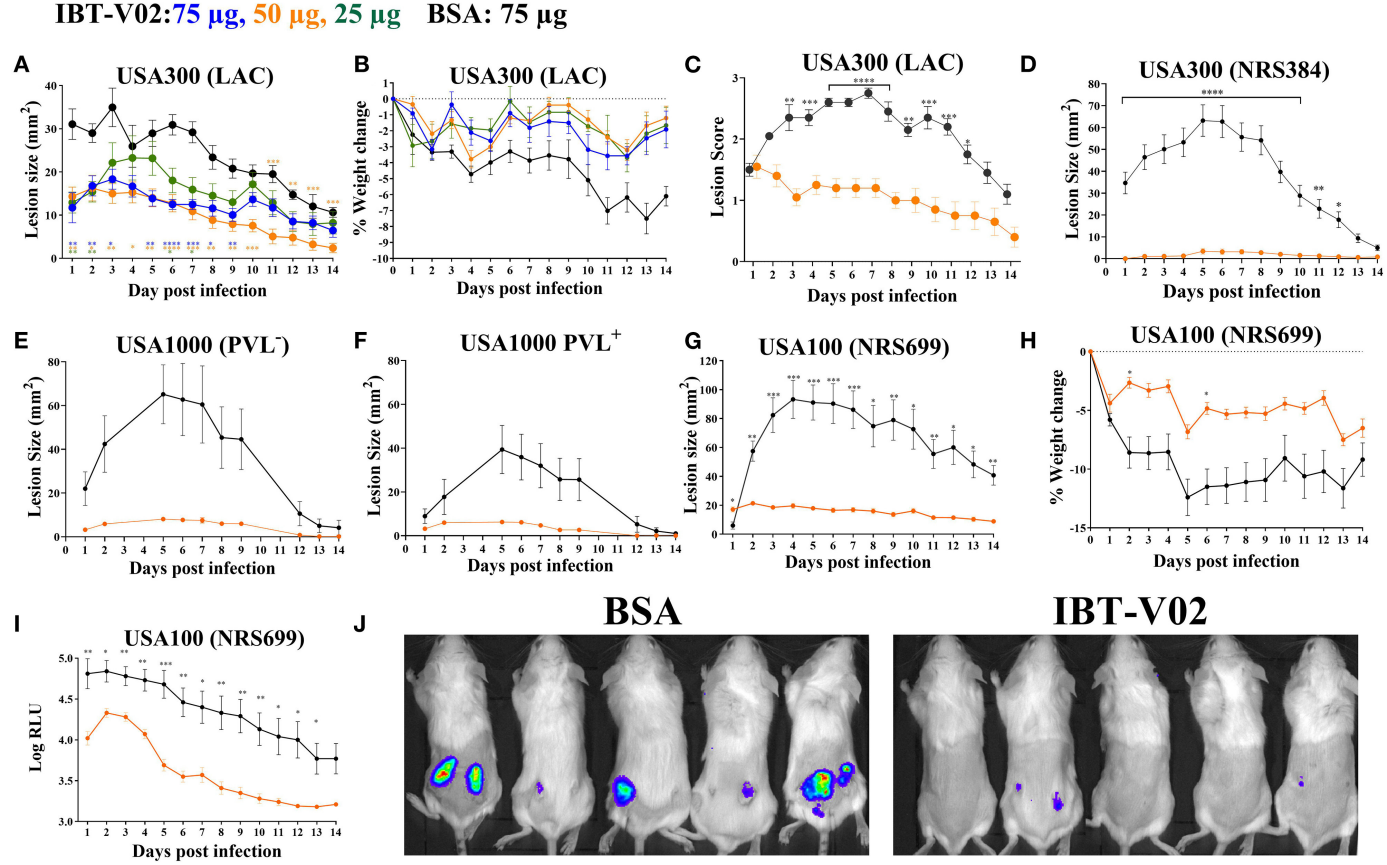
Efficacy of IBT-V02 in mouse skin infection. **(A–C)** Efficacy of IBT-V02 at 75, 50, and 25 μg doses against skin infection with USA300 (LAC), *n* = 5/group. **(A)** Lesion size, **(B)** weight loss and **(C)** lesion scores measured for 14 days. **(D–J)** Efficacy of IBT-V02 vs. BSA at 50 μg/mouse. Lesion size after infection with **(D)** USA300 (NRS384), *n* = 10/group, **(E)** USA1000 PVL^−^, *n* = 5/group or **(F)** USA1000 PVL^+^, *n* = 5/group. **(G–J)** Efficacy of IBT-V02 at 50 μg/mouse against luminescent USA100 (NRS699), *n* = 10/group. **(G)** Lesion size, **(H)** weight loss, **(I)** relative light units (RLU) in skin as a readout of bacterial burden, and **(J)** bioluminescent imaging of USA100-infected mice on day 7 post infection. Data were analyzed using GraphPad PRISM v8.4.3. Statistical analysis for lesion size performed using 2-way ANOVA, Sidak's multiple comparisons test.

To test the efficacy of IBT-V02 in an acute skin infection model, groups of 10 mice were vaccinated with 75, 50, or 25 μg of the vaccine candidate, or 75 μg of BSA as control with the same schedule described above. Mice were challenged with 1 × 10^7^ CFU of USA300 (LAC) on day 42 by subcutaneous (SC) infection and were monitored for 14 days for lesion size, weight change, and any sign of disease. All mice vaccinated with 50 or 75 μg of IBT-V02 showed significantly smaller lesions than control animals at all time points (Figure 3A). The efficacy was also reflected in reduced weight loss (Figure 3B) and lower lesion severity scores (Figure 3C).

The efficacy of IBT-V02 (50 μg/mouse) was further tested using another USA300 isolate (NRS384), a USA100 clone (NRS699) transduced with *lux* operon, as well as two USA1000 isolates. IBT-V02 showed remarkable efficacy against all strains as measured by lesion size, weight loss, or bacterial burden measured as luminescence intensity (Figures 3D–J).

### Protection Is Driven by Vaccine-Generated Serum Antibodies

**Figure 4:**
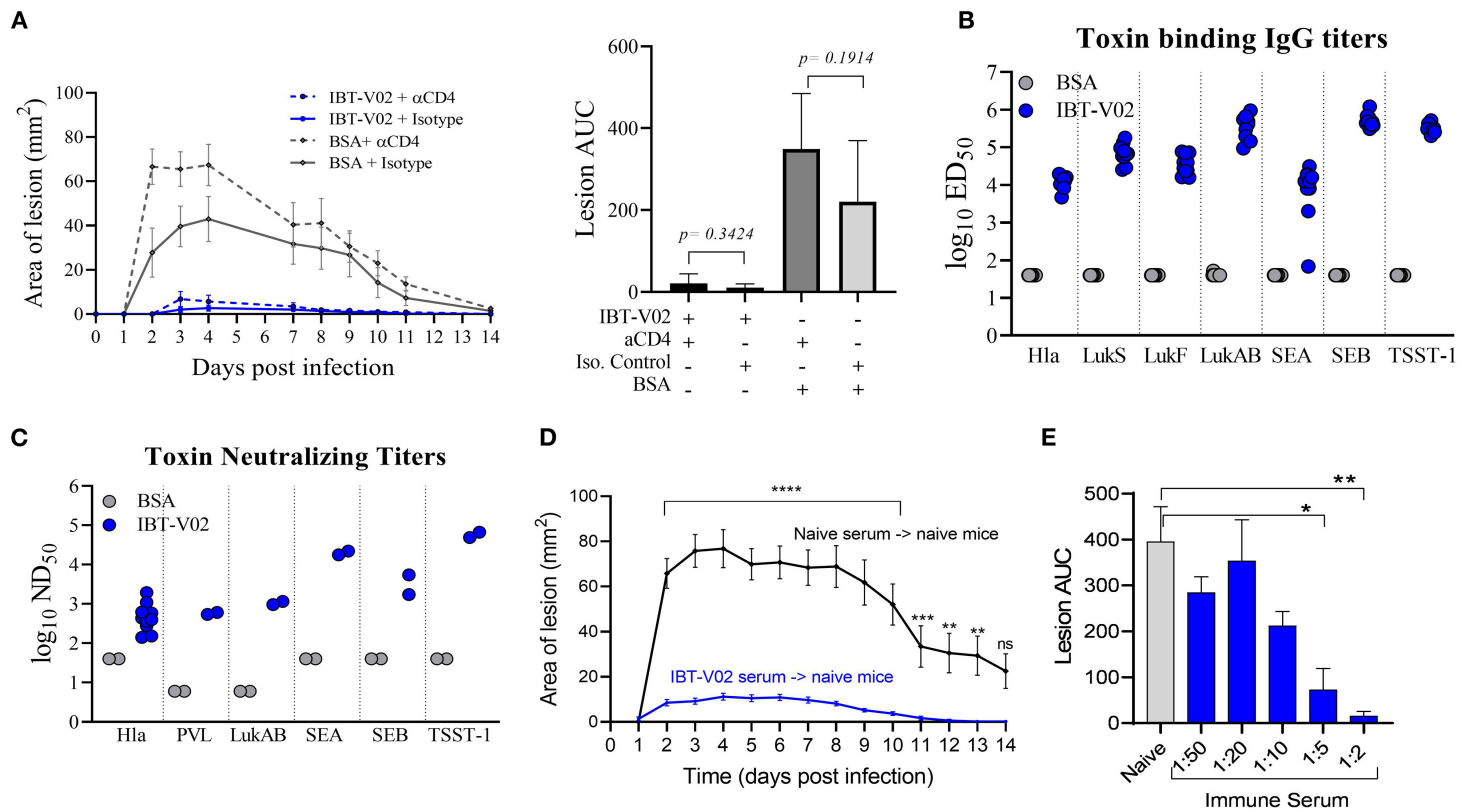
Protection against USA300 (NRS384) is mediated by vaccine-generated serum antibodies. **(A)** Lesion size and area under the curve after depletion of CD4 T cells in BSA-immunized and IBT-V02 immunized mice during infection, *n* = 5/group. **(B)** Toxin binding IgG titers tested on individual serum samples. **(C)** Toxin neutralizing titers tested on individual serum samples against Hla, and titers tested on serum pools/group against PVL, LukAB, SEA, SEB, and TSST-1 prior to CD4 T cells depletion. **(D)** Lesion size after passive transfer of naïve or immune sera previously generated in CD1 mice into BALB/c mice, *n* = 10/group. Statistical analysis performed using two-way ANOVA, Sidak's multiple comparisons test. **(E)** Area under the curve of infection-induced lesions after passive transfer of naïve sera or different dilutions of immune sera. Statistical analysis was performed by *t*-test using GraphPad PRISM v8.4.3. Siginificance symbols: *P ≤ 0.05; **P ≤ 0.01; ***P ≤ 0.001; ****P ≤ 0.0001.

To evaluate a potential protective role of vaccine elicited CD4 T cells, mice were immunized with either IBT-V02 or BSA, and CD4 T cells were depleted 72 h prior to and 48 h post-infection with 1 × 10^7^ CFU of USA300 (NRS384) using an anti-CD4 monoclonal antibody (mAb clone GK1.5). Mice treated with isotype control antibodies were used as controls. As shown in Figure 4A, mice treated with anti-CD4 mAb showed lesions similar in size to mice treated with isotype control antibodies, indicating that the protective efficacy of IBT-V02 in mice was not driven by vaccine generated CD4 T cells. Interestingly, depletion of CD4 T cells in mice immunized with BSA resulted in larger lesions when compared to isotype treated BSA control. However, areas under the curve for mouse lesions showed no significant differences between these two groups (Figure 4A). Sera collected 2 weeks after the last immunization were characterized for toxin binding IgG titers (Figure 4B) as well as toxin neutralizing antibodies (Figure 4C). All mice demonstrated robust IgG titers toward all target antigens, with the exception of one mouse that showed low titers toward SEA (Figure 4B). Individual serum samples tested against Hla and serum pools tested against remaining target antigens all showed robust toxin neutralizing antibody titers (Figure 4C). Next, we evaluated the protective role of vaccine-generated serum antibodies by passive serum transfer studies. Immune sera were generated in ICR (CD1) mice through repeated immunization (4x 2-weeks apart). Sera were collected, pooled, and characterized for toxin-specific IgG binding titers (Supplementary Figure 1A) and toxin neutralizing titers (Supplementary Figure 1B). Serum pools demonstrated high toxin binding as well as neutralizing antibody titers against all target antigens (Supplementary Figures 1A,B). Naïve BALB/c mice were then treated with 500 μl of neat IBT-V02 immune sera intraperitoneally (IP) 4 h prior to intradermal infection with 1 × 10^7^ CFU of USA300 (NRS384). Sera collected from naïve mice were administered to control animals, and the treatment was repeated 4 days post-infection. Lesions were monitored and measured for 14 days. As shown in Figure 4D, mice treated with immune sera developed significantly smaller lesions when compared to mice treated with naïve sera. The potency of the vaccine generated serum antibodies was highlighted even more when immune serum was diluted in PBS prior to treatment and still showed protective efficacy at 1:2 and 1:5 dilutions (Figure 4E).

**Figure 5:**
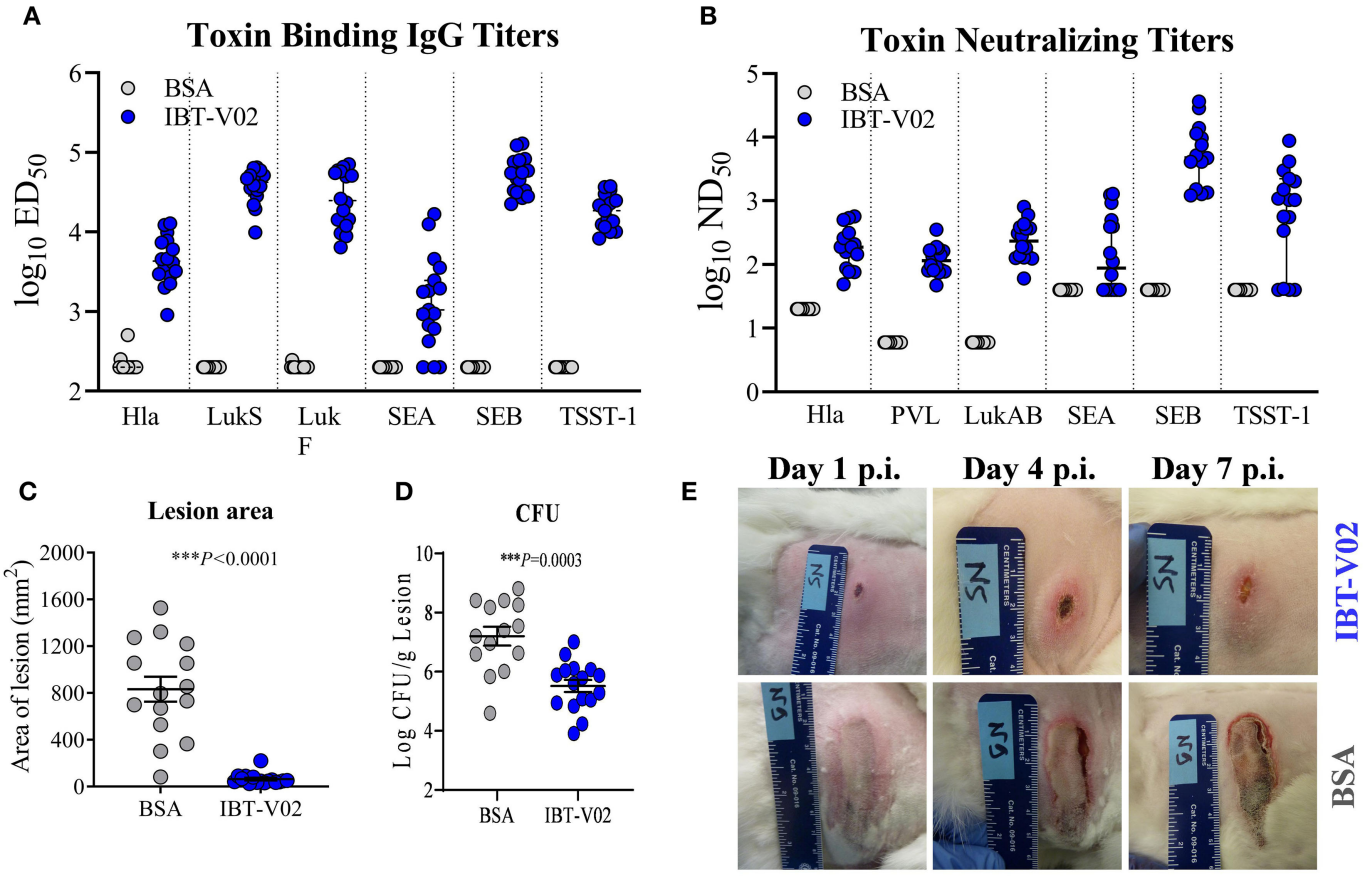
Efficacy of IBT-V02 in a rabbit dermonecrosis model with USA300 (NRS384). **(A)** Serum IgG binding titers and **(B)** Serum toxin neutralizing titers of rabbits immunized with BSA or IBT-V02 tested against target antigens Hla, PVL (LukS & LukF), LukAB, SEA, SEB, and TSST-1. **(C)** Lesion area and **(D)** bacterial burden on day 7 post infection **(E)** Lesion images of BSA or IBT-V02 immunized rabbits on days 1, 4, and 7 post infection. Data analyzed using GraphPad PRISM v8.4.3. Statistical analysis performed with Mann-Whitney test. Shown are mean values with SEM.

The immunogenicity as well as the efficacy of IBT-V02 was further evaluated in a rabbit model of acute skin infection. Two groups of sixteen New Zealand White rabbits (NZWR) were immunized ID three times 2 weeks apart with 100 μg of IBT-V02. Rabbits were infected ID with 9.94 × 10^9^ CFU of MRSA USA300 (NRS384), and the lesions were monitored for 7 days post-infection and pictures were taken daily. High total IgG titers (ED_50_) were achieved against TSST-1, SEB, SEA, Hla, LukS-PV, LukF-PV, and LukAB (Figure 5A). Immunization of rabbits also induced highly significant neutralizing titers toward the target antigens of IBT-V02 as well as cross neutralizing activity toward closely related bi-component pore forming toxins HlgAB Figure 5B) and HlgCB, consistent with previous findings (27). Lesions of rabbits immunized with IBT-V02 were significantly smaller (Figure 5C) and had lower bacterial burden (Figure 5D) on day 7 post-infection compared to controls. Figure 5E shows representative images of lesions on days 1, 4, and 7 post-infection.

### Pre-exposure to *S. aureus* Is Not Protective and Does Not Impact IBT-V02 Efficacy Against SA-ASI

**Figure 6:**
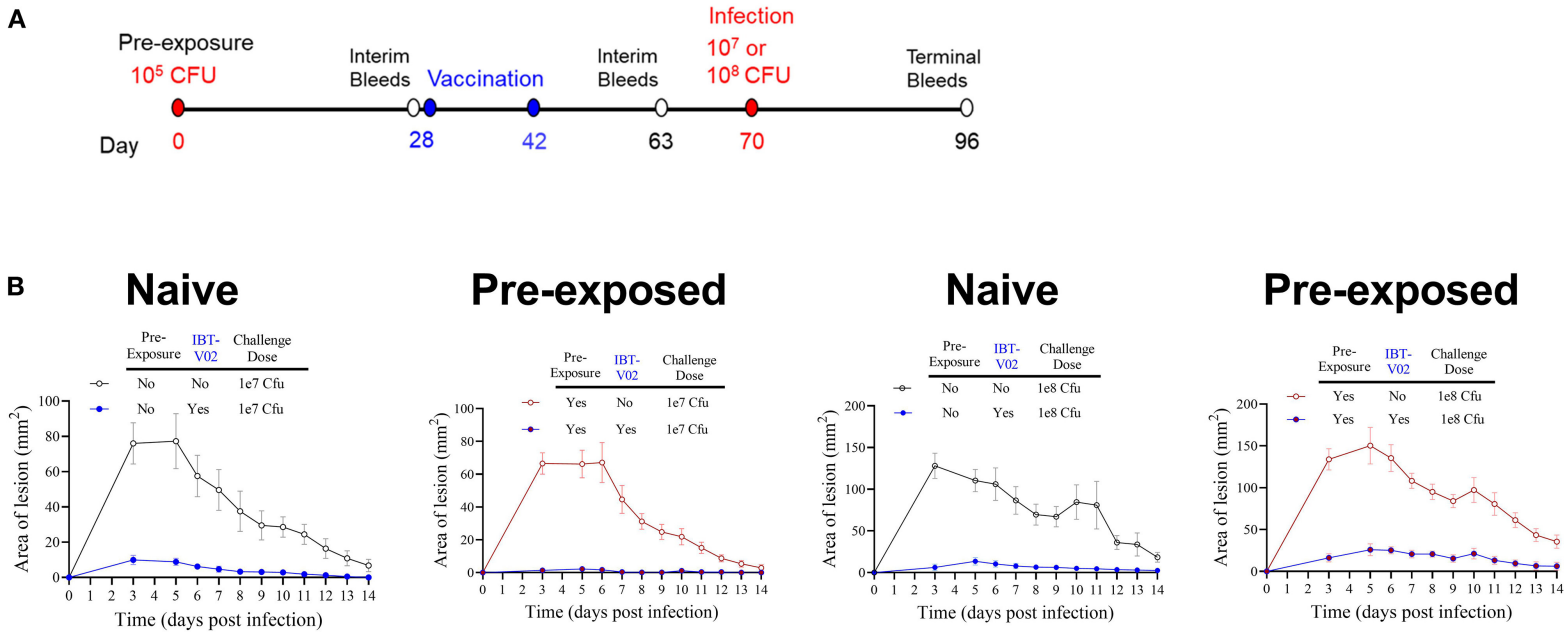
Efficacy of IBT-V02 in pre-exposed mice. **(A)** Infection and vaccination schedule. **(B)** Lesion areas of mice with or without intradermal pre-exposure prior to infection with 10^7^ or 10^8^ CFU of USA300 (NRS384), *n* = 5/group, shown are mean values with SEM.

Pre-clinical evaluation of vaccine candidates is commonly performed in naïve animals, and this has been the case for SA vaccine candidate vaccines evaluated to-date. However, contrary to mice, most humans are not naïve for SA since a large part of the population is persistently or intermittently colonized by SA and demonstrate pre-existing memory against a wide range of staphylococcal antigens (45–48). Therefore, we evaluated whether the efficacy of IBT-V02 in mice was affected by cutaneous pre-exposure to SA. As shown in Figure 6A, groups of 10 mice were intradermally injected with (pre-exposed to) 1 × 10^5^ CFU of SA USAS300 (NRS384) or left untreated as a control on study day 0. The animals were then vaccinated with IBT-V02 on days 28 and 42. Animals were rested for an additional 4 weeks and then challenged ID with either 1 × 10^7^ or 1 × 10^8^ CFU. Mice were monitored for another 2 weeks for lesion size and general health. Mice pre-exposed to SA exhibited the same course of lesion development and healing as the naïve animals (Figure 6B), indicating that cutaneous pre-exposure to SA neither provides protection against subsequent infection nor has an impact on vaccine efficacy. Similarly, IBT-V02 protective efficacy was comparable between naïve and pre-exposed animals.

### Vaccination During Acute Phase of Infection Protects Against Secondary Infection

**Figure 7:**
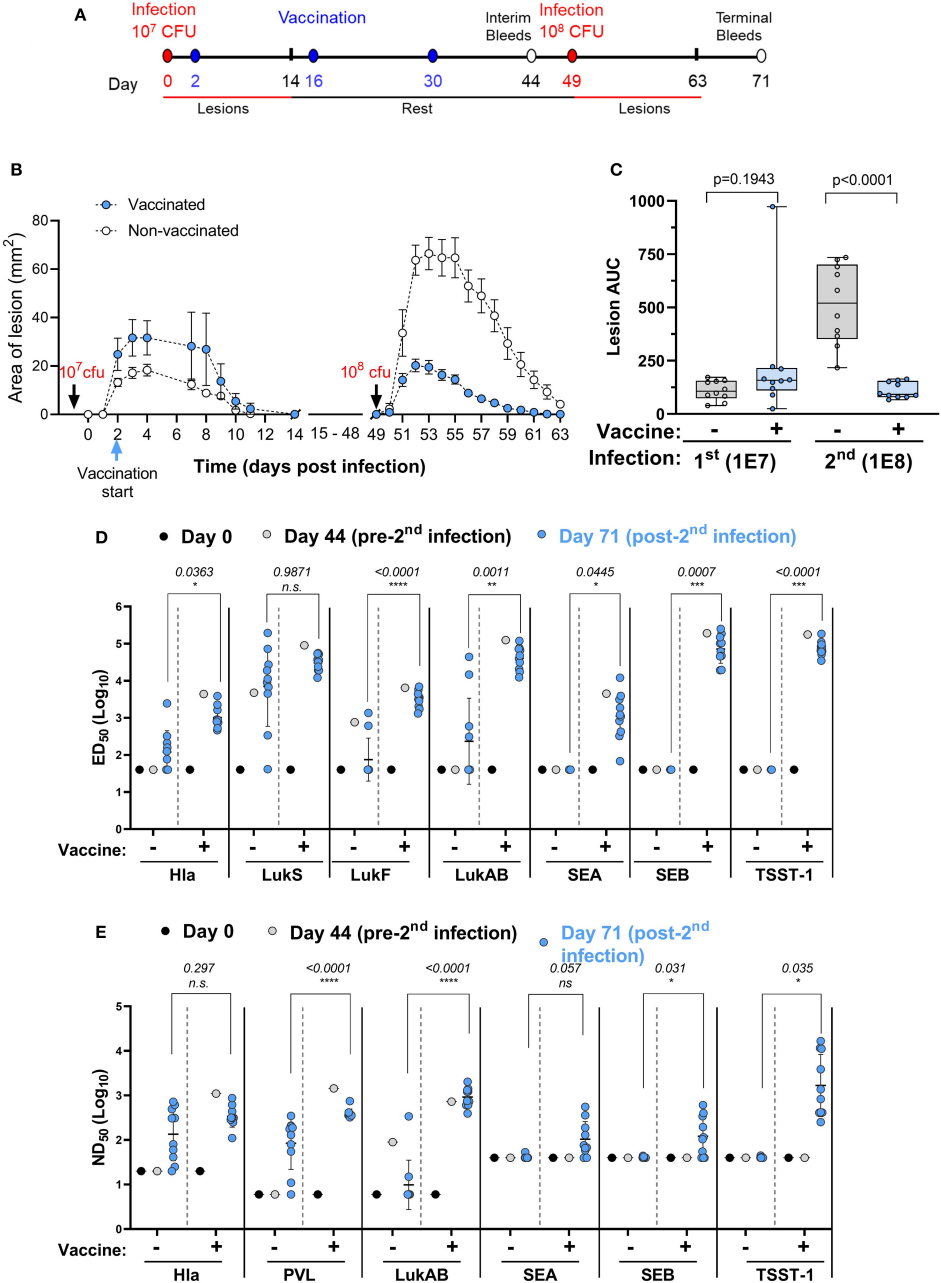
Efficacy of IBT-V02 during active infection. **(A)** Infection and vaccination schedule. **(B)** Lesion areas of vaccinated and non-vaccinated mice after primary (10^7^ CFU) and secondary infection (10^8^ CFU) with USA300. **(C)** Area under curve of infection-induced lesions of individual mice. **(D)** Serum IgG binding titers with lower limits of detection (LLOD) at 40, and **(E)** Serum toxin neutralizing titers with LLOD of 20 (Hla), 6 (PVL & LukAB), and 40 (SEA, SEB, TSST-1) of vaccinated and non-vaccinated mice on serum pools collected on days 0 and 44 and on individual serum samples collected on day 71 tested against target antigens Hla, PVL (LukS & LukF), LukAB, SEA, SEB, and TSST-1, *n* = 10 mice/group. Data were analyzed using GraphPad PRISM v8.4.3. Statistical analysis was performed using *t*-test. Shown are log-transformed mean values with SD.

Recurrence of SA skin infections is frequently seen and an important public health problem. A vaccine that could protect against recurrence is therefore of high value. The ideal time to vaccinate individuals against recurrence may be during the active infection. Here we asked the question whether immunization started in the acute phase of a primary infection can protect against secondary infection with a higher challenge dose. Two groups of 10 mice were infected intradermally with 1 × 10^7^ CFU of SA USA300 (NRS384) on study day 0. One group received IBT-V02 (IM) on study days 2, 16, and 30 while the other group served as infection only control. Both groups were infected with 1 × 10^8^ CFU on day 49 and monitored for another 14 days. Animals were bled on days 0, 44, and 71 for serological analysis. The study design is shown in Figure 7A.

As shown in Figure 7B, vaccination during acute infection did not affect the lesion size as further supported by the AUC analysis (Figure 7C). After the second infection with a high dose of USA300, the control animals exhibited much larger lesions than the primary lesions (Figures 7B,C), further confirming the findings in our previous experiment (Figure 6). However, animals vaccinated during the acute phase exhibited highly significant reduction in lesion size compared to non-vaccinated mice (Figures 7B,C).

We further measured the total IgG and neutralizing titers against the toxins represented by IBT-V02 on serum pools collected from mice on days 0 and 44 and on individual serum samples collected on day 71. The first infection in non-vaccinated mice induced very low levels of total IgG against Hla and LukAB and moderate levels of antibodies against LukS-PV and LukF-PV (Figure 7D, day 0 vs. day 44 in the no vaccine groups). However, the second infection in the no vaccine group did boost the antibodies against pore forming toxin antigens Hla, LukS-PV, and LukAB, but not LukF-PV (Figure 7D, day 44 vs. day 71 in the no vaccine groups). Vaccination induced high IgG titers against all antigens and the antibody levels were not significantly changed after the second challenge (Figure 7D, see the vaccine groups, day 44 vs. day 71). In all cases, except for LukS, the day 71 titers (post-second challenge) were significantly higher in the vaccinated than in non-vaccinated mice (Figure 7D). The primary infection failed to induce any appreciable neutralizing titers against Hla and PVL, but the titers were moderately increased after the second infection, whereas the modest LukAB neutralizing activity seen in the serum pool remained low after the second infection (Figure 7E, the no vaccine groups). Vaccination during infection induced high neutralizing activity against all three cytolysins which was retained upon high dose reinfection (Figure 7D). Whereas, vaccinated groups demonstrated robust IgG binding titers (Figure 7D) and neutralizing titers (Figure 7E) against SEA, SEB, and TSST-1, serum antibodies generated against these SAgs were not detected in the no vaccine group (Figures 7D,E), which was not surprising as USA300 (NRS384) does not harbor any of these SAgs.

## Discussion

*S. aureus* (SA) is the most common cause of purulent acute skin infections in humans, leading to a high burden of disease in both healthy and immunocompromised individuals with an estimated 8.7 million ambulatory visits alone in the US (30, 49, 50). SA also complicates burn and surgical wound sites and lesions of atopic dermatitis (51, 52). A major problem for patients with acute skin infection is the high rate of recurrence that can be as high as 70% in 1 year (41, 53–55). Recurrence is hardly reduced by successful treatment of the primary lesion using incision and drainage and/or antibiotics (56). SA produces a wide range of cytolytic and superantigenic toxins with a wide range of pathogenic and immune subversive activities severely affecting the course of disease including skin infections (20, 21, 35). Here we present an entirely toxoid multi-component SA vaccine (IBT-V02) targeting Hla, PVL, LukAB, and three superantigens (SEA, SEB, and TSST-1) and demonstrate its immunogenicity and protective efficacy in mouse and rabbit models of dermonecrosis. We demonstrate efficacy of the vaccine in both naïve mice and mice pre-exposed to SA as well as when administered during acute infection.

The role of cytolytic toxins in the pathogenesis of SA skin infections is well-documented through human epidemiological studies and animal models (57). Prominent among these toxins is Hla, which is the main driver of the skin dermonecrotic lesions caused by SA in mouse and rabbit models (35, 58). Hla is highly hemolytic for rabbit red blood cells but not for human erythrocytes (59), and its main pathogenic activity in humans is likely due to its ability to damage the skin and mucosal barriers by targeting epithelial cells (60–63), endothelium (64, 65), and keratinocytes (66), as well as pro-inflammatory properties through inflammasome activation (35). Hla also contributes to biofilm formation by SA wound isolates (67). Furthermore, Hla causes platelet aggregation that can contribute to micro-thrombi leading to organ failure (68). Hla monoclonal antibodies or vaccines are protective against dermonecrosis in mice (69, 70). In humans, antibodies against Hla have been shown to correlate with a better clinical outcome in patients with SSTI (71). PVL, a prominent toxin produced by the epidemic clone USA300 and a growing number of strains around the world (72), is implicated in the epidemiology of SSTIs. PVL was linked to community-acquired MRSA (CA-MRSA) outbreaks including SA skin infection in the 1900s (2). In a meta-analysis, the presence of the *pvl* gene was associated with abscesses and furuncles (73). Whereas, wild type mice do not respond to PVL, in non-obese diabetic (NOD)/severe combined immune deficiency (SCID)/IL2rγ^null^ (NSG) mice reconstituted with human umbilical cord blood cells and administered fresh human neutrophils, a 1–2 log lower USA300 inoculum is needed to induce consistent skin lesions compared to an isogenic PVL-negative strain (37). Consistently, the PVL-negative SA strain induced smaller lesions compared to the parental strain (37). Both PVL and LukAB cause dose dependent skin inflammation in rabbits (74). Staphylococcal superantigens (SAgs) have been implicated in colonization of skin with SA and the etiology of atopic dermatitis (AD), a chronic disease that pre-disposes patients to recurrent SA skin infections. The majority of SA isolates from atopic eczema produce SAgs (75), and SAgs are likely a major factor in the Th2 type inflammatory response in AD patients (34). Consistent with this, SAgs facilitate epithelial presentation of allergens to Th2 cells (76). Adhesion molecules that bind to SA, such as fibronectin and laminin, are also upregulated as a result of the Th2 cytokine IL-4 released in the inflammatory environment of skin (77). IL-4 and IL-13 have also been shown to suppress IFNγ- or TNFα-induced expression of antimicrobial peptides in keratinocytes (77). Thus, neutralization of superantigens in the skin is expected to change the immunological environment to unfavorable conditions for colonization and infection. Thus, an effective vaccine for SA-ASI should target these cytolytic toxins as well as major superantigens.

The 5-component vaccine IBT-V02 presented here includes toxoids of Hla, both PVL subunits, LukAB as well as a fusion of toxoids for TSST-1, SEB, SEA formulated in Alhydrogel (Al(OH)_3_). All individual components of the vaccine were characterized and published previously and shown to be highly attenuated and to induce broad neutralizing antibody responses (24–27). Here we show that the blended vaccine formulated with Al(OH)_3_ is highly immunogenic in mice and rabbits, inducing a strong total IgG and neutralizing antibody response. We tested the vaccine for efficacy against dermonecrosis caused by SA-ASI with several clinically relevant strains of CA-MRSA (ST-8), USA300 and USA1000 (ST-59), and USA100 (ST-5) clones. IBT-V02 showed protective efficacy against all the strains tested with highly significant reduction in lesion size, lesion score, weight loss, and bacterial burden. The protective efficacy in this model was primarily mediated by serum antibodies as depletion of CD4 cells at the time of infection in vaccinated or control animals had no significant impact on the vaccine efficacy, whereas adoptive hyperimmune serum transfer to naïve animals was highly protective.

About two thirds of the human population is persistently or intermittently colonized with SA and, as a result, most people have immunological memory toward staphylococcal antigens. Yet most vaccine candidates have been tested in immunologically naïve animals. To evaluate whether pre-exposure has an impact on the efficacy of IBT-V02, we exposed naïve mice to a low dose of USA300 by the cutaneous route, similar to the most common route of human exposure, before vaccination with IBT-V02 and challenge. The results of this study demonstrated that (i) pre-exposure to SA does not elicit a protective immune response, and (ii) vaccine efficacy is identical between naïve and pre-exposed animals.

Recurrent SSTI is a major public health problem, a burden on the families of patients, a significant driver of healthcare costs, and a driver of antibiotic use, thus contributing to antibiotic resistance (40). Some patients can experience repeated bouts of recurrent infections over months and even years after primary infection. Given that ~0.4% of patients with SA skin infections develop systemic infections (56), recurrence further increases the risk of invasive disease. While patients with comorbidities are generally considered more prone to recurrence, a major portion of recurrent skin infections are in patients with no major comorbidities (56). To address the issue of recurrent infections, we developed a re-infection model in mice using two subsequent intradermal infections with increasing challenge doses. Our data show that not only an asymptomatic exposure, but even a full-blown, symptomatic primary infection, does not protect against SA re-infection.

Our data further show that IBT-V02 can be administered during the acute phase of the first infection to protect against a subsequent reinfection. Mice that were vaccinated during the first infection showed significantly less severe lesions upon a second challenge with a ten-fold higher dose. This is important because patient compliance with requests to return for vaccination against a possible future recurrence could present a roadblock to prevention, especially when the primary infection is not severe or life threatening. Offering the vaccine at the time of infection for diseases with the risk of recurrence, such as *SA* SSTI or bacteremia, *C. difficile* infections, or urinary tract infections, could significantly increase vaccine coverage and reduce the burden of disease.

In summary, we have presented here the first fully toxoid multi-component vaccine against acute and recurrent SA skin infections. Importantly, we have demonstrated efficacy of the toxoid vaccine against a secondary skin infection. Future studies are needed to further characterize the immune response that is critical for protection against secondary infection including the role of the immune response against superantigens in more SAg-sensitive animal models as well as in future clinical trials. Our findings along with a body of evidence on the role of anti-toxin antibodies in improvement of clinical outcome of SA disease [reviewed extensively in (57)] strongly support testing of this vaccine for prevention and mitigation of recurrent SA-ASI in human clinical trials.

## Materials and Methods

### SDS-PAGE, Western Blot, and Size Exclusion High Performance Liquid Chromatography

IBT-V02, composed of 5 components representing 7 toxoids, was characterized by SDS, WB, and SE-HPLC. For WB, antigen-specific primary and secondary antibodies were used. For SEC-HPLC, 10–40 μg of each IBT-V02 component was injected in an Agilent Technologies 1260 Infinity Series instrument using an AdvanceBio SEC 300 Å 7.8 × 300 mm LC column with a mobile phase of 50 mM sodium phosphate buffer + 150 mM NaCl, pH 7.0, running at a flow rate of 0.5 mL/min. The chromatogram generated by the Agilent OpenLabs software plotted absorbance at 280 nm as a function of retention time. All analyses of the peaks were performed by the auto-integrate function in the OpenLabs software.

### Adsorption of IBT-V02 to Alhydrogel

IBT-V02 was incubated with Alhydrogel (Brenntag Bio) at various antigen: aluminum ratios for 1 h at room temperature. After incubation, the antigen-adjuvant mixture was centrifuged, and the supernatant was run on an SDS-PAGE gel. Adsorption of antigens to Alhydrogel was indicated by a thin or negligible band of protein visible on the gel as compared to a control without Alhydrogel adsorption, depicted by the non-adsorbed complete antigen band.

### Bacterial Strains

SA strains USA300 (LAC), USA300 (NRS384), were obtained from the BEI resources. Strain USA300 (SF8300) and USA1000 (ST59, PVL^+^) (78) was provided by Dr. Binh Diep at UCSF. Strain USA1000 (PVL-) was obtained from a repository collected from patients with SA sepsis (79). Bioluminescent NRS699 strain (NRS699 lux) was generated in house: Strain SAP140, an RN4220 strain with pRP1195 plasmid (Temperature sensitive plasmid with Lux ABCDE cassette) was received as a gift from Dr. Roger Plaut, FDA (80). The SAP140 phage lysate was made at 30°C using 80α phage. The transduction was carried out at 30°C to move the temperature sensitive plasmid into recipient NRS699 strain (Gift from Dr. Jean C. Lee) using a standard transduction protocol (80). Integration of plasmid into the recipient chromosome was carried out by shifting the growth temperature to 43°C in the each transductant strain (80). Three clones were screened under IVIS camera to confirm the bioluminescent phenotype.

### Preparation of Inoculum for Infection

For mouse skin infection with USA300 (LAC) and USA100 (NRS699) bacteria were incubated at 37°C, shaking at 200 rpm in tryptic soy broth (TSB) to mid-exponential phase. The culture was centrifuged and washed twice with PBS prior to injection. Inoculum was verified by CFU/ml determinations. For mouse skin infection with USA300 (NRS384) and USA1000, Brain-Heart Infusion (BHI) broth was inoculated with a swab of SA from a freshly grown blood agar plate, and culture was grown for 18 h at 37°C, shaking at 230 rpm. The culture was centrifuged at 3,000 rpm for 10 min, and the pellet was washed twice in PBS before suspensions in PBS. To ensure single cell suspension, bacterial preparations were passed through a syringe with a 27-gauge needle before freezing aliquots at −80°C until further use. For the rabbit ABSSSI model, USA300 (NRS384) was prepared as previously described (81).

### Animals, Vaccinations and Infection

Female Balb/C mice were purchased from Charles River. The starting age of mice for each experiment was 6 weeks. Mice were maintained under pathogen-free conditions and fed laboratory chow and water ad *libitum*. Mice were immunized intramuscularly on each side of the tail base (50 μl each side) three times 2 weeks apart with a total of 50 μg of IBT-V02 (10 μg each antigen) in 250 μg Alhydrogel. For serological analyses, the mice were bled *via* the retro-orbital (RO) or tail vein route prior to and 10–14 days after the final immunization. Two weeks after the last immunization, the backs of the mice were shaved and 50 μl of *S. aureus* suspension was administered *via* intradermal (ID) or subcutaneous (SC) injection. The animals were monitored daily for weight loss. Lesions were measured daily for 14 days post-infection using calipers. The areas of the lesions were calculated using the formula Area (A) = Length (L)/2 x Width (W)/2 x π. Statistical significance was determined using two-way ANOVA with Sidak's multiple comparisons analysis (GraphPad PRISM v8.4.3). As described in the text, some experiments required two immunizations after pre-exposure or during acute infection. For passive serum transfer studies, female CD1 mice, 8 weeks of age were purchased from ENVIGO and were immunized four times 2 weeks apart with 50 μg of IBT-V02 formulated in 250 μg Alhydrogel. Terminal bleeds were performed 2 weeks after the final immunization, and pooled sera from these animals were stored at −80°C until further use. Pooled sera from naïve CD1 mice purchased from ENVIGO served as controls. Mice were maintained under pathogen-free conditions and fed laboratory chow and water ad *libitum*. All mouse work was conducted in accordance with protocols that were approved by institutional animal care and use committees (IACUC) of Integrated BioTherapeutics, where mouse studies were performed.

Animal experiments performed at the Brigham and Women's Hospital were conducted in accordance with the recommendations and guidelines in the Public Health Service Policy on Humane Care and Use of Laboratory Animals, and animal use protocols were approved by the Partners Healthcare Institutional Animal Care and Use Committee. Mice that were challenged subcutaneously with luminescent USA100 (NRS 699) were monitored daily for weight loss and lesion size. Mice were imaged with an IVIS Lumina 3 system, and bioluminescence was quantified with Living Image 4.7 software.

The rabbit ABSSSI model was reviewed and approved by the University of California San Francisco Institutional Animal Care and Use Committee. Experiments were conducted in a facility certified by the Association for Assessment and Accreditation of Laboratory Animal Care International. Female and male New Zealand White (NZW) rabbits were purchased from Western Oregon Rabbit Co. The starting age range of rabbits for each experiment was 8–11 weeks. Rabbits were immunized intradermally on the right and left dorsal lumbar skin three times 2 weeks apart with a total of 100 μg of IBT-V02 (20 μg each antigen) in 500 μg Alhydrogel. Animals were bled prior to immunization to obtain naïve sera and 2 weeks after the last immunization to obtain immune sera. The vaccinated rabbits were challenged in the ABSSSI model as previously described (81).

### Serum Total Antibody Titers

A multiplex assay to detect serum IgG titers to SA antigens has been previously developed at IBT using the Luminex xMAP® technology. Briefly, IBT-V02 target antigens Hla, LukS-PV, LukF-PV, LukAB, SEA, SEB, and TSST-1 were coupled to carboxylated MagPlex microsphere beads with distinct spectral regions *via* a carbodiimide reaction. Antigen-coupled beads were incubated with serum samples at a starting dilution of 1:40 in a two-fold 8-point dilution series at room temperature (RT) for 2 h. Samples were washed and incubated with a PE-conjugated goat anti-mouse IgG antibody (Biolegend, San Diego, CA.) for 1 hour at RT. The samples were washed and acquired using a Luminex200. Data were analyzed using a 4-parameter (4PL) curve fit in XLFit (Microsoft). IgG titers were expressed as the effective dilution at the point of the 4PL curve where 50% (ED_50_) of antigen was detected by toxin-specific antibodies present in the serum sample.

### Serum Total Neutralizing Titers

Hla TNAs were performed as previously described (82). In brief, 4% rabbit red blood cells (RRBCs) were co-cultured with wild-type Hla ± serially diluted serum samples. Cells were centrifuged after 30 min and absorbance determined at OD_416nm_. PVL and LukAB TNAs were performed with human promyelocytic leukemia (HL-60) cells as previously described (25). In brief, differentiated HL-60 cells were incubated with either PVL or LukAB ± serially diluted serum samples for 3 h, and CellTiter Glo was added to the culture to measure cell viability. SAg TNAs were performed with PBMCS from healthy volunteers. Cells were co-cultured with SEA, SEB, or TSST-1 in the presence or absence of serially diluted serum samples for 48 h, supernatants were collected, and IFNγ was measured in the supernatants as a readout of superantigenicity as we previously described (26). Data were analyzed using a 4-parameter (4PL) curve fit in XLFit (Microsoft). Toxin neutralizing activity was defined as the effective dilution of sera at the point of the 4PL curve at which 50% of toxin activity was neutralized (ND_50_).

## Data Availability Statement

The raw data supporting the conclusions of this article will be made available by the authors, without undue reservation.

## Ethics Statement

The animal study was reviewed and approved by Integrated Biotherapeutics IACUC.

## Author Contributions

HK, AV, JL, BD, and MA designed experiments. IM, MM, RO, NN, AV, RA, TK, and JL carried out experiments and analyzed data. JL, BD, MA, FH, and HK interpreted data. HK, AV, and MA wrote manuscript. MA acquired funding. All authors contributed to the article and approved the submitted version.

## Conflict of Interest

MA and HK have stocks and RA and FH have stock options in Integrated Biotherapeutics Inc. JL serves on the Scientific Advisory Board of Integrated BioTherapeutics. The remaining authors declare that the research was conducted in the absence of any commercial or financial relationships that could be construed as a potential conflict of interest.
