# Supplementary material for: IBT-V02: A Multicomponent Toxoid Vaccine Protects Against Primary and Secondary Skin Infections Caused by Staphylococcus aureus
Source: Front Immunol. 2021 Mar 10;12:624310. doi: 10.3389/fimmu.2021.624310 (PMC7987673; doi:10.3389/fimmu.2021.624310)
Supplement: Supplementary file 1 [file Data_Sheet_1.PDF]

Supplemental Fig 1

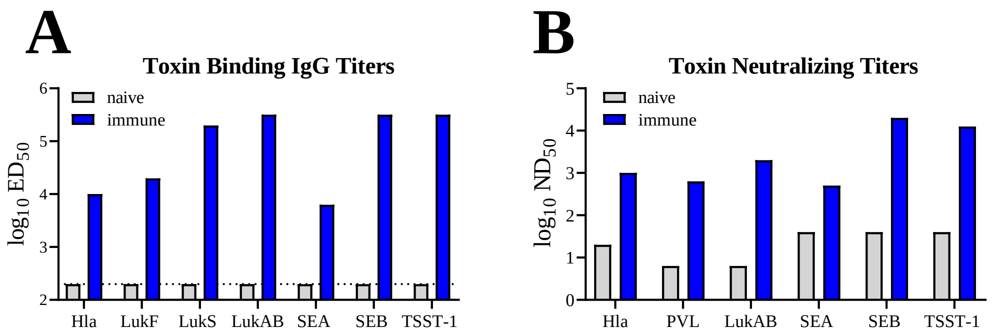

S1. Generation of immune sera against IBT-V02 in CD1 mice. (**A**) Toxin binding IgG titers and (**B**) toxin neutralizing titers of serum pools from CD1 mice immunized three times 2-weeks apart.
